# Assessment of Food Quality and Safety of Cultivated Macroalgae

**DOI:** 10.3390/foods11010083

**Published:** 2021-12-29

**Authors:** Trond Løvdal, Dagbjørn Skipnes

**Affiliations:** Nofima—Norwegian Institute of Food, Fisheries and Aquaculture Research, Department of Process Technology, Richard Johnsens Gate 4, P.O. Box 8034, NO-4021 Stavanger, Norway; dagbjorn.skipnes@nofima.no

Macroalgae aquaculture is 16 times larger than fish on a mass basis, making macroalgae by far the largest group of aquacultured products. Still, both the macroalgae market and aquaculture are underdeveloped around the world except for Asia, in particular China. Macroalgae are an important source of minerals, iodine, vitamins, and polyunsaturated fatty acids, and these nutrients are important for their beneficial effects on human health. As a low trophic food or food ingredient from the sea, with an enormous potential for increase, macroalgae will play an increasing role in the sustainable circular bioeconomy. Despite the rising popularity of Asian cuisine and the Western consumers’ perception of seaweeds as a “healthy superfood”, understanding consumer behaviour in relation to new foods and facilitating information-based decisions could reduce potential consumer scepticism. Thus, this Special Issue focuses on seaweeds for food and feed purposes, with emphasis on the food quality and safety aspects.

From a consumer perspective, a food product should first of all be appealing, i.e., look, smell, and taste well. This comes naturally with seaweeds, and this (re-)discovered culinary ingredient has trigged the creativity of many chefs and product developers. However, more research is needed on sensorial properties of seaweeds depending on species, location, cultivation mode, seasonality, part of the plant, and processing conditions [1]. Rapid post-harvest deterioration of seaweeds can be avoided through stabilisation techniques, for instance, through temporary storage solutions before final processing, direct utilisation of food items, and packaging. Innovative drying and alternative processing strategies may reduce energy consumption and processing time while at the same time improving the safety as well as the nutritional and sensory qualities of the product.

Fermentation is an enabling and clean technology that effectively stabilises the seaweed raw material and, at the same time, can be used to modify flavour, texture, and acceptability of seaweeds, and to improve their functional and nutritional properties [1]. Successful fermentation stabilises the raw seaweed biomass by producing lactic acid and quickly reducing the pH of the seaweeds, thereby inhibiting the growth of pathogenic bacteria. The studies of Akomea-Frempong et al. [2] revealed that blanching, freezing, and fermentation had positive impacts on kelp quality and consumer acceptability. They demonstrated a positive effect of blanching on the greenness and firmness of sugar kelp (*S. latissima*) salad, and they also showed that blanching followed by freezing had positive sensory effects in fermented sugar kelp/cabbage sauerkraut and, at the same time, improved food safety. The possibility to use frozen seaweed as an ingredient in sauerkraut may also increase retail availability throughout the year and mask some aroma notes of kelp such as pungency and fishiness when used to develop products [2]. Salting is another effective measure to preserve seaweeds. Wei et al. [3] evaluated the effects of salt concentrations (10, 20, 30%) at 25 and 4 °C on microbial diversity and spoilage. They found that a salinity above 20% preserved several seaweed species even at room temperature storage, and refrigeration preservation at a salinity of 10% worked as well [3]. However, the high salt content may limit the further use in several products and the amount of seaweed that can be used without further processing. Sanchez-Garcia et al. [4] evaluated chemical, physical, and sensory changes in the green seaweed *Ulva rigida* during storage at 4 and 16 °C. The quality of *U. rigida* was better preserved at refrigeration, slowing down both the enzymatic and microbial activity responsible for the deterioration, compared with 16 °C. The pH, the percentage of exudate, and the changes in colour and texture indicate a loss of seaweed freshness correlated with the increase in microbial cells and the sensory analysis [4]. The shelf-life of raw *U. rigida* was documented to be one week when stored at 4 °C, but the quality changes limit the culinary use [4].

The nutritional potential of seaweeds is very interesting. The polyphenol of brown and green macroalgae is very high, while red macroalgae have high protein content. All seaweed species have a high content of fibre and minerals, and the fatty acid composition is very beneficial with a very low omega-6/omega-3 ratio [5]. The nutritional quality of seaweed in terms of vitamin C was reviewed by Nielsen et al. [6]. Seaweed is a large and diverse group, and this comprehensive review summarises how 92 different seaweed species contribute to daily vitamin C intake. The review concludes that seaweeds are not a rich source of vitamin C, but when consumed they contribute to the daily intake. The vitamin C content is very species dependent and is also variable, depending on biological, seasonal, locational, and treatment variations. It was found that drying, boiling, and long storage time lead to a decrease in vitamin C in seaweed, as it is easily oxidised [6].

Tackling safety issues related to human consumption of seaweeds is required for their widespread use in food applications. Sustainable, multi-target mitigation strategies towards microbiological and chemical (excessive iodine, heavy metals, allergens) hazards are driving the improvement of food safety of seaweeds and their derived products [7]. Seaweed food safety in terms of iodine and heavy metals content, allergenicity, microbiology, etc. has been reviewed previously [8,9]. A comprehensive review on food safety focussing entirely on the microbiological factors was provided by Løvdal et al. [10]. The review identified (i) seaweed-associated pathogenic bacteria that are often present in the marine environment and on the surface of seaweeds and, thus, are of special concern for food safety; (ii) viruses and several bacterial species that are considered as potential food safety concerns, predominantly by virtue of recontamination during processing and handling; (iii) other pathogenic microorganisms that can, on rare occasions, lead to food poisoning but particularly because of gross violations of food safety protocols, including break of cooling regimes, or bad quality water conditions at harvest or cultivation sites. The review also summarised processing technologies and other measures to control microbial growth and thus ensure microbial food safety, and legislations and guidelines relevant to edible seaweeds, and data gaps requiring further research were pointed out [10]. The relatively high content of iodine and heavy metals in some seaweed species, and especially in the brown macroalgae, limits the use of seaweeds as a food source for human consumption because it sometimes exceeds permittable levels and thus may contribute to the accumulation of unhealthy and toxic compounds in the body. The effect of cultivation depth on the content of potentially toxic elements in *Saccharina latissima* was investigated in the paper of Blikra et al. [11]. Interestingly, seaweeds cultivated at 1 m depth contained less iodine than those cultivated at 6 or 9 m. However, after processing the difference was no longer significant. Cultivation depth did not have any significant effect on the content of arsenic, cadmium, lead, or mercury [11]. Technologies intended to reduce the content of heavy metals and iodine include ultrasound-assisted approaches as investigated by Noriega-Fernandez et al. [12]. The potential of ultrasound (US), alone or in combination with mild heat treatment and/or EDTA towards reduction of As, Cd, I, and Hg in *Laminaria hyperborea* was evaluated. The combined application of US, mild heating, and EDTA led to 32%, 52%, and 31% release of As, Cd, and I, respectively, from *L. hyperborean*, thus significantly improving the products’ food safety for consumers [12]. However, most of the reduction could be achieved by heat treatment alone, while US treatment was less important. It is likely that this could prove right for kelp used for food as well.

Red seaweeds are seen as an alternative cattle feed in order to reduce greenhouse gas (i.e., methane) emission from ruminants because they contain halogenated compounds including bromoform that decrease such emission. However, bromoform is toxic and thus potentially harmful to cattle and to humans consuming cow milk if it is excreted to milk. Muizelaar et al. [13] investigated the rate of transfer of bromoform from red seaweed *Asparagopsis taxiformis* and *Asparagopsis armata* fed to dairy cows to their milk, urine, faeces, and animal tissue, and to gain more insight into the effects of feeding red seaweed on animal health, ruminal changes, feed intake, milk production, and milk composition. Results showed that feeding the cows red seaweeds reduced feed intake. Within the confines of the experiment, bromoform did not accumulate in the cows’ tissue but was excreted in milk and urine when fed *A. taxiformis* containing 1.26 mg/kg dry matter of bromoform. Abnormalities of rumen wall papillae were also observed in the cows fed *A. taxiformis* [13]. The authors concluded that more research is needed to define the long-term effects of red seaweed on the rumen wall and of the presence and metabolism of bromoform in milk and urine [13].

In the Special Issue “Assessment of Food Quality and Safety of Cultivated Macroalgae”, both quality and safety have been discussed from several viewpoints, and the innovative tools discussed in this Special Issue can be exploited for further development of a sustainable seaweed food industry. The major food safety issues reported are high levels of iodine, arsenic, and heavy metals for some species aquacultured at some locations. This does not exclude other threats, e.g., microbial contamination. However, it has also been shown that there are effective measures that in many cases will guarantee food safety for seaweed products.

## Data Availability

Not applicable.
